# Replantation of Teeth

**Published:** 1873-11

**Authors:** Isidor I. Lyons


Replantation of Teeth.—*Isidor I. Lyons, M.R.C.S.*—Replantation of teeth, as a useful remedy in dental surgery, has never been received with much favor by the dental profession. The results of cases prove, however, that at least there are conditions in which it is of great value.

The principal objection urged against replantation of teeth is, that if a tooth is extracted, it must necessarily lose its vitality, and therefore the fangs undergo absorption, so that after a time it becomes useless, and must be extracted. Supposing the objection to be valid, as absorption is a long process, sometimes extending over years, it will have been a greater pain for a patient to retain his tooth for an indefinite period than to lose it entirely and at once; but it is no more necessary that a tooth, after undergoing extraction and replantation, should lose its vitality, than for a long bone to do so after fracture, with stripping back of the periosteum. The extraction of a tooth may be divided into two parts: firstly, the laceration of the alveolo-dental membrane or periosteum; secondly, that of the nerve and blood-vessels supplying the tooth. As regards the former, there is no reason why the alveolo-periosteum should not again unite to the tooth, seeing that if a piece of periosteum be stripped off a bone, it will unite again if placed in contact with the bone and left at rest. The union of the divided ends of a nerve is also a recognized fact; but even supposing this latter impossible, the tooth would merely be in the condition of one which has had its pulp destroyed—a common operation in dental surgery.

The manner of performing the operation is as follows: A tooth which is to be replanted should be carefully extracted, and as little as possible of the surrounding tissues lacerated; it should then, unless the operation be simply for the destruction of the dental pulp, and where the periosteum is healthy, be immersed in some antiseptic fluid, such as diluted carbolic acid or chloride of zinc (the latter, from experience, being preferred); the socket should then be swabbed out some half-dozen times with a strong solution of the same antiseptic employed. The tooth, if carious, should be plugged and returned to its place. If there is any thickening of the periosteum, fibrous growth, sac of abscess, or absorption at the extremity of the fang, it should be excised before replantation. Should patient complain of pain arising frnm the operation, prescribe poppy fomentations, although the pain is rarely more than what is due to the tenderness of parts from the laceration of soft tissues after extraction of the tooth.

Out of twelve cases that I have operated on within the last four years, nine are successful, and three have failed. The failures have but one significance, and that is, teeth to undergo replantation must be selected. In a cachectic patient the chances are against success; when a tooth has lost the support of its fellows on both sides, it cannot become firm. Nevertheless, the successful cases warrant a further trial of replantation, which would preserve many teeth otherwise sacrificed. The number of my cases during the last four years were twenty-seven, some of which cannot be traced; the remainder are too recent to be judged. The reason why they are so few arises from the difficulty of inducing patients to believe in such a remedy as replantation.

